# Medication Self-Management for Home Care Users Receiving Multidose Drug Dispensing: Qualitative Interview Study

**DOI:** 10.2196/57651

**Published:** 2024-10-04

**Authors:** Anette Vik Josendal, Trine Strand Bergmo

**Affiliations:** 1 Norwegian Centre for E-health Research, University Hospital of North Norway, Tromsø, Norway; 2 Department of Pharmacy, University of Oslo, Oslo, Norway; 3 Department of Pharmacy, The University of Tromsø – The Arctic University of Norway, Tromsø, Norway

**Keywords:** home care, medication management, adherence, self-management, multidose drug dispensing, Norway, primary care, older adults

## Abstract

**Background:**

Multidose drug dispensing (MDD) is an adherence aid where medicines are machine-dispensed in disposable unit bags, usually for a 14-day period. MDD replaces manually filled dosettes in many home care services in Norway. While evidence suggests that MDD can improve medication adherence and reduce errors, there are few studies on how patients manage MDD at home and how this affects their daily routines.

**Objective:**

The aim of the study is to identify factors influencing medication self-management behavior among MDD users living at home and explore how MDD affects medication self-management.

**Methods:**

We conducted semistructured interviews with 19 MDD users in Oslo between August 2019 and February 2020. The interviews were held at the participants’ homes, and the interview transcripts were analyzed thematically.

**Results:**

All participants in the study received some form of assistance with medication management from home care services. This assistance ranged from MDD delivery every other week to actual assistance with medication administration multiple times daily. However, regardless of the level of assistance received, participants primarily managed their MDD medications themselves. Daily medication routines and knowledge about medicines varied among the participants, with some taking an active role in their medication management, while others relied on others to take responsibility. The degree of involvement seemed determined by motivation rather than capability.

**Conclusions:**

MDD can support medication self-management, but its effectiveness varies among patients. The level of medication management by MDD users is not solely determined by their actual capabilities. Factors such as interest in self-care and independence, available support, information, and cognitive capacity all play a role in determining the degree of autonomy.

## Introduction

Medications play a crucial role in modern medicine. However, various studies have indicated that patients encounter drug-related problems and struggle with medication management [1-5]. Medication nonadherence, when medications are not taken as prescribed, has significant consequences, leading to poorer health outcomes and increased costs for society [5].

To adhere to a medication regimen, patients must undertake a series of actions that demand specific knowledge, skills, and behavior. According to the model developed by Bailey et al [6], medication self-management can be categorized into 6 phases: filling the prescription, understanding how to take the medications correctly, organizing the medication use, taking the medication, monitoring effects and side effects, and sustaining use. These phases necessitate various skills, and numerous factors can contribute to patients being unable to manage their medications correctly. For instance, the complexity of the medication regimen, impaired vision and manual dexterity, polypharmacy, medication knowledge, perceptions about the severity of one’s illness, and experiences of side effects are among the contributing factors [7]. Additionally, there are medication-related risk factors, such as confusion between generic and brand names, lack of medication administration routines, hoarding, retaining discontinued medication, and involvement of multiple prescribers [8].

In Norway, as in several other countries, there is now a trend toward shorter hospital stays due to an increased focus on providing health and care services in patients’ homes [9,10]. Consequently, more individuals with medication management problems are living at home with help from home care services. One of the main goals of the service is to enable individuals to live in their homes for as long as possible, both to improve the quality of life and to contain costs for the health system. One area of support often provided by home care nurses is to help with medication dispensing and administration. In Norway, about one-third of home care users get help administering their medications through multidose drug dispensing (MDD) [11].

MDD is a dispensing system where solid medications are machine-dispensed in unit-of-use disposable bags, 1 bag for each dose occasion [12]. The bags are labeled with patient information, name and strength of the medications, and date and time of day the medications should be taken. Only solid medications such as tablets and capsules can be dispensed as MDD. Although MDD systems are common in many hospitals across the world where the system has shown to reduce certain types of medication errors, only some countries use it in primary care [13]. The MDD system has the potential to reduce medication costs, improve medication adherence, and reduce medication errors also in a primary care setting [12,14]; however, like with other adherence aids, the scientific evidence to support these claims is limited [12,15-18]. In general, there are few studies on how patients manage MDD at home and how this affects their daily routines [12,17-19].

In this study, we aim to explore how MDD is used by patients living at home and how the service affects medication self-management and to identify factors influencing medication self-management behavior.

## Methods

### Study Design

A qualitative approach with face-to-face, semistructured, in-depth interviews was used. We used the 32-item checklist of COREQ (Consolidated Criteria for Reporting Qualitative Research) for the reporting of this study [20].

### Setting

All residents in Norway have a legal right to home care services provided by municipalities [9]. In 2022, approximately 172,000 citizens in Norway received home nursing services, with about one-third receiving help with administering medications through MDD services [11].

Municipalities purchase the MDD service from 2 main suppliers in Norway. The packaging fee varies between municipalities due to tender prices, but each municipality can be reimbursed 500 NOK (US $47) per MDD user per year [21]. Home care services are responsible for selecting patients for the MDD service, although it is recommended that both the patient and their general practitioner (GP) are involved in the decision [22]. Once a patient starts MDD, the pharmacist creates an MDD “prescription card” that includes a complete list of prescribed medications, regular medications (both dispensed as MDD and in their original packaging), as-needed medications, medical devices, and dietary supplements. MDD bags are usually dispensed every 2 weeks, while other medications are dispensed in their original packaging from a local pharmacy.

MDD bags do not include package inserts, but the MDD supplier provides a copy of the prescription card, which includes dosing schedules and short descriptions of each medication’s indication for use. They also provide pictures and descriptions of the dispensed medications, including instructions on whether tablets should be swallowed whole, split, or crushed. Some MDD users have automated dispensers for their MDD bags, which can remind them when to take medications and notify home care services if medications are not taken. However, most users manage their medications directly from the MDD bags without dispensers.

### Recruitment

Due to privacy reasons, recruitment had to be done via the home care service. The study recruited MDD users from 4 different home care districts in Oslo, targeting both high and low socioeconomic areas. A nurse from each district contacted users and asked if they could give their phone numbers to the first author (AVJ) for participation in this study. The nurses were asked to recruit users with varying levels of independence. The inclusion criteria were MDD use, being 18 years or older, and able to consent. Of 25‐40 users per district, a total of 21 agreed to be contacted by the researcher (AVJ), 19 ultimately participated in the interview study. The researchers did not know the nurses or informants prior to the study.

### Data Collection

An interview guide (Multimedia Appendix 1) was developed by both authors, guided by findings from previous research [23,24]. The 2 main topics of the interview guides were the use of medications and information about medications. Before the interview started, the users were also asked to put their medications in clear view, where appropriate. The interview guide was pilot-tested on 2 MDD users in November 2018, which led to minor changes in the guide. These pilot interviews were not included in the results of this study.

All interviews were completed by the first author (AVJ) in the user’s home. Two interviews had 2 participants (spouses, where both used MDD), the remaining were individual interviews. The interviews lasted from 26 to 86 minutes. Fifteen were recorded on tape and transcribed verbatim by one of the researchers (AVJ). In the remaining 2 interviews, the participants did not want to be audiotaped, and AVJ wrote down the dialogue by shorthand. Immediately after these interviews, AVJ repeated the interviews for herself based on the notes and memory. After completing approximately 15 interviews, no new data emerged; however, 2 additional interviews were made to ensure data saturation [25]. The study was conducted from October 2019 to February 2020.

### Authors’ Preunderstanding

The 2 researchers (AVJ and TSB) had different backgrounds providing different perspectives. AVJ is a pharmacist, and at the time of the study, a PhD student in social pharmacy. She has worked at a community pharmacy, providing MDD to nursing home patients, and at an MDD manufacturer for many years prior to this study. TSB is a senior researcher in health service research, with previous 7 years of clinical experience as a registered nurse. Both authors have some experience (3‐4 studies) with qualitative research.

### Data Analyses

The transcripts were coded manually in Microsoft Word (Microsoft Corp). Both authors read, discussed, and structured the transcribed material and participated in the analysis of the data. To identify factors influencing medication self-management, we followed the 6 steps of thematic analysis as described by Braun and Clarke [26]. This resulted in the themes and subthemes shown in the Results section.

### Ethical Considerations

This study was approved by the data protection officer at the University Hospital of North Norway (project 02003). Participants were recruited by a home care nurse due to privacy reasons. The participants received both written and oral information, including information about the length of the interviews, anonymity of responses, and data management, before signing an informed consent form. The interviews were labeled with a study identification number and not the informant’s name. During the transcribing, names of persons or places were deleted, and the recordings were deleted after all interviews were transcribed.

## Results

### Overview

In total, 19 informants were interviewed. The majority (n=14) were female, the age range was from 59 to 92 years (Table 1). All the participants in this study had assistance with medication management from home care. For some, this only included getting the MDD delivered every other week and no other assistance, while for others this included help with both administering medications and other daily living activities several times a day. Two of the users got MDD dispensers.

**Table 1.** Characteristics of respondents.

ID	Age (years)	Sex	Home care visits
#1	76	Female	3 per day
#2	62	Female	1 per day
#3	88	Female	2 per month
#4	82	Female	3 per day
#5	71	Male	1 per week
#6	59	Female	2 per day
#7a	88	Male	1 per day
#7b	89	Female	1 per day
#8	87	Female	3 per day
#9	87	Female	1 per day
#10	92	Female	1 per day
#11	82	Male	2 per week
#12	66	Male	2 per month
#13	91	Female	1 per day
#14	72	Female	1 per day
#15	83	Female	2 per day
#16	62	Female	2 per day
#17a	82	Male	3 per day
#17b	82	Female	3 per day

**Figure 1.**
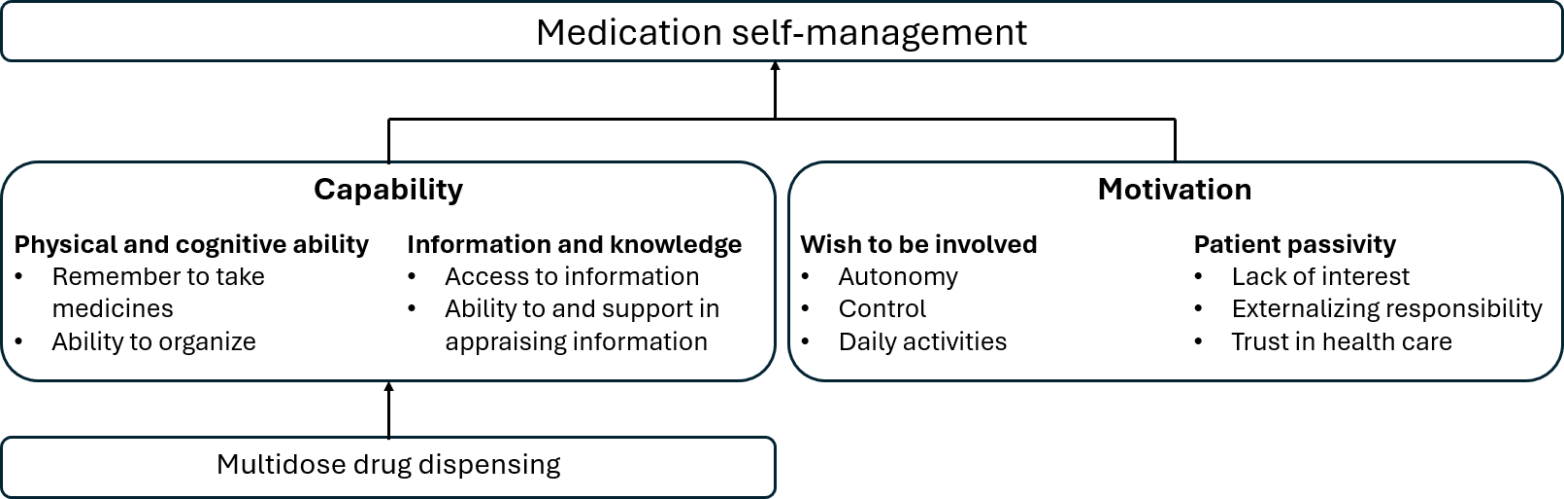
Main themes and subcategories of factors influencing medication self-management.

There was a high degree of variation in the informants’ daily medication-taking routines as well as interest and knowledge about medications. We identified four main themes that influence the MDD user’s medication self-management at home: (1) physical and cognitive ability, (2) information and knowledge, (3) wish to be involved, and (4) patient passivity (Figure 1).

### Physical and Cognitive Ability

Participants in the study highlighted the convenience of having MDDs delivered to their homes, eliminating the need to keep track of prescriptions. One participant cited this as the reason for starting MDD, as she was no longer able to walk to the pharmacy to collect her medications and had no other issues with managing them. While some participants faced challenges with opening the bags and experienced tablets falling out; overall, they expressed satisfaction with the MDD system, finding it easier to take medication correctly and appreciating the clear instructions provided.

*Many old people have declining vision, and it’s very easy to make mistakes, but when you have one of these [MDD bags] you cannot make mistakes. Everything is written here, the time to take them and everything [you need]. So, this is one of the best things they [home care] have ever started.*

[Participant #15]

Mental capabilities were also discussed as a crucial factor in medication management. Organizing medication use was often cited as a challenge, leading participants or their families to request MDD services when they felt they could no longer safely keep track of their medications. Participants expressed relief in having one less thing to worry about in their daily lives when their medications were organized in the MDD bags.

*I’m very happy with the MDD (...). Here it says the time and date and everything. Every day, so I don’t have to think about it. And the dosing is correct too I assume because it’s pharmacists who’ve done it.*

[Participant #5]

While the MDD system did not provide reminders for medication intake, most participants had established routines that helped them remember to take their medications. They linked medication intake to daily activities, such as eating breakfast. Despite the assistance provided by the MDD system in organizing medication use, participants emphasized the importance of personal capacity and the potential for mistakes even with the use of adherence aids like MDD.

*You know, when you read about things that has happened with medications. Maybe someone has got something they’re not supposed to. Imagine that you’ve started forgetting, that can easily happen. If they have dosette boxes, that doesn’t help. You can make mistakes with dosettes.*

[Participant #13]

### Information and Knowledge

The level of information and knowledge about medication use varied among the study participants. Some had a comprehensive understanding of their prescribed medications, including the reasons for taking them and the correct dosage. However, others had limited knowledge and were unsure why they were taking certain medications. The desire for more information did not necessarily correlate with the participant’s level of knowledge. Even those with minimal knowledge expressed satisfaction with the information they received and did not express a desire to learn more.

*I do get it [information] sometimes. A printed thing. But like I said, I’m not interested in it. I do have it around here somewhere, but I don’t really know what’s going on (...) I at least hope they [the medicines] don’t harm me.*

[Participant #4]

While most participants were aware of the prescription card that accompanied their MDD bags, many did not actively use it to keep track of their medications. Some participants found the MDD system helpful because it provided them with information about the names of their medications, which they struggled to remember before. However, there were also participants who felt that the MDD system made it harder for them to maintain an overview of their medications. One participant mentioned that they used to fill their own dosette boxes, which gave them a better understanding of their medications, but after transitioning to the MDD system, they felt they had lost control and struggled to keep track of what they were taking.

Participants expressed difficulties in obtaining the information they desired about their medications. Only a few participants actively used computers or the internet to access information. Some participants missed the information leaflets that were included with medications dispensed in their original packaging, while others desired more information on the prescription card provided with the MDD bags. Some participants felt that it was not just the lack of information itself that was challenging but also the lack of time to discuss their medications with their health care providers and receive help in understanding the information they had received.

*I have asked [the home workers] many times. You can just read this, but that doesn’t help me, I need help to understand it.*

[Participant #16]

### Wish to Be Involved

The patients who actively participated in the medication management process often expressed a wish or felt the need to be involved in the process. Organizing medication use and taking medications were the 2 steps they were mostly engaged in, but also to some extent seeking information about their medications. One informant phrased this as an active decision that one had to make:

*You have to want to (...) people can’t stand outside of your door telling you they will help you, you have to do something yourself as well. (...) Things don’t come by itself. That’s how it is with people, we tend to take the easiest way out.*

[Participant #15]

Despite receiving medications in the form of MDD, participants still had to establish routines to integrate medication intake into their daily schedules. Many viewed this as the primary reason to engage in medication self-management. Several participants described how they adjusted their medication schedules based on their daily plans and activities, either by bringing the MDD bags with them or modifying the timing of medication intake. Some mentioned receiving assistance with afternoon medications only, as home care services were not available early enough for morning medication help.

Additionally, participants made adjustments to their medication routines based on their health-related needs. For instance, one participant described how she every evening opened the MDD bag for the next morning and took out the painkillers from the bag. She placed these on the nightstand so she could take them as she woke up, while the rest of the medications in the bag she left by the breakfast table to be taken a few hours later.

The desire to maintain independence and a sense of control over their situation motivated some participants to engage in self-management. For them, being able to perform tasks they were capable of themselves, rather than relying entirely on home care workers, was important for their independence and self-esteem.

*They [home care workers] would prefer to apply the plaster (...) but I do not like being dependent on them doing it. I tell them that I manage it myself, and they have accepted that.*

[Participant #10]

For users who had problems with organizing medications, the need to be involved manifested as routines to double-check the home care workers’ work or the content of the MDD bags. This included actions such as sorting the tablets into egg glasses or visually inspecting and counting tablets before taking them.

### Patient Passivity

All participants stated that they were capable of managing their MDD medications on their own, even those who required assistance with non-MDD medications such as inhalers, creams, or eye drops. However, not all participants took on this responsibility. One participant explained that while she was capable of taking her medications, she believed it was the home care worker’s responsibility to provide them, and she only took the MDD bags herself if the home care workers had forgotten to give them to her during their visit. Another participant expressed a lack of interest in learning more about her medications, as she did not see medication management as her responsibility.

*I take the medicines I am given. If anything goes wrong it is not my fault.*

[Participant #4]

In many instances, the lack of engagement in medication self-management seemed to be related to a high level of trust in the GP or the MDD system. Participants expressed a significant amount of trust in their GPs, relying on their decisions and not questioning their prescribing practices. Most participants had been seeing the same GP for a long time and found it easy to contact them if they needed a new prescription or had questions. Given this trust in the GP and the MDD system, participants did not perceive a need to be actively involved in the medication management process.

*No, I don’t know. I take [the medicines] they tell me to take. As long as they’re in the multidose bags I don’t think more about it. And as you see here, there are pictures of all the tablets.*

[Participant #2]

### How the MDD System Affects Medication Self-Management

Many of the informants described how the MDD system helped them manage their medications, particularly with removing the need to keep track of prescriptions and help in organizing use. Based on the model of medication self-management by Bailey et al [6], the MDD system, however, affected all 5 initial phases of medication self-management:

- The first step in medication self-management—filling prescriptions and keeping track of prescriptions—was eliminated by the MDD system.
- In terms of understanding how to take medications, the MDD system appeared to reduce patients’ ability to keep track of their medications, especially for those who desired and were capable of actively participating in their medication management. On the other hand, for patients who were less interested in self-care, the MDD system had either no impact or a positive one, as it reduced the amount of information they had to process.
- In terms of organizing medication use, the MDD system generally facilitated the organization of medication intake. However, participants still needed to adjust their timings and routines to accommodate their daily schedules.
- When it came to taking medications, the participants reported good overall adherence. However, there were instances of both intentional and unintentional medication nonadherence. The MDD bags provided clear instructions for the safe administration of medications, but they did not inherently help patients remember to take their medications.
- Finally, in terms of monitoring, the absence of information leaflets accompanying the MDD bags seemed to reduce users’ knowledge and, consequently, their ability to monitor the effects of their medications. This lack of information posed a potential drawback to the monitoring phase of medication self-management.

## Discussion

### Principal Findings

This study explored how MDD users living at home self-manage their medications. All the participants received assistance with medication management from home care. Some participants only got their MDD delivered every other week, while others received help multiple times a day. The level of engagement in medication management varied greatly among patients, primarily dependent on their motivation rather than their actual capabilities. The MDD service showed a positive impact on patient’s ability to keep track of and filling prescriptions and on medication organization. However, the service could also adversely affect patient’s ability to monitor their medications and decrease their knowledge about them.

### Motivation to Participate

Perhaps one of the most obvious factors we found influencing medication self-management was the informants’ preferences and motivation for involvement in the process. This came across as a choice informants made, which was not necessarily related to their capabilities of self-management. The reason for wanting to be involved could differ, and the users were thus not necessarily motivated and interested in being involved in all the steps. For instance, individuals who wanted to be involved for the purpose of fitting medication management into their daily schedules were not necessarily motivated to learn more about their medications or how to use them correctly.

In contrast, other participants clearly stated that they were not interested in participating and were happy with simply “following the doctor’s orders.” This type of “passive” medication user, who trusts their GP and with little interest of more information about their treatment, has also been described in previous studies [27-31]. There seems to be a correlation between increasing age and lower desire for participation [20,31], and in our study, most participants were older patients. The high degree of trust in the doctor’s medical expertise is a predictor of medication adherence [21]. However, to accept information without question can also be a sign of inadequate health literacy, which again is associated with poorer medication adherence [22,32].

Patient preferences, while important, may not necessarily indicate how safely patients are managing their medications. Patients relying on their doctor’s decisions can still make informed health choices, while those solely relying on their own judgment may make risky decisions [33]. The key lies in recognizing when to act autonomously and when to seek guidance from a physician [21].

### Physical and Mental Capabilities

Poor cognition is associated with poor adherence and medication management capacity [7,34]. Informants in our study also stressed the importance of cognitive abilities in relation to medication self-management. Some had started the MDD service because of a decline in cognitive abilities and not being able to dose their medications safely anymore. However, even with adherence aids, such as MDD, informants described that cognitive abilities were still important to be able to manage these safely. This illustrates that even though MDD can support users in parts of medication management, it does not support all the phases of the process [6,35].

It is important to note that patients’ capabilities and motivations to manage their medications are not always directly connected. Some capable patients choose not to manage their medications, while others who struggle still want to be involved and develop routines to maintain control, for example, sorting medications into other containers, inspecting or counting tablets, or intentionally adjusting their medication intake to fit their daily schedules. Although such alterations have previously been described as potentially reducing the safety of the MDD system and increasing the risk of medication errors [12,23,28], our participants described them as a way to regain control after transitioning to the MDD system, and in such a way, have a positive effect on their autonomy.

For some patients, MDD solved physical problems, such as difficulty walking to the pharmacy, but questions remain about whether MDD is the right tool for patients with such issues. A previous study from the Netherlands has shown that about 30% of patients with MDD, despite having some challenges, might not have lost their capacity to manage medications [36]. This raises concerns about the appropriateness of the MDD system, especially considering potential negative impacts on other phases of medication self-management.

### Knowledge of Medications

Knowledge about medications is crucial for patients’ ability to manage them [6,21,33]. However, studies have shown that MDD users often have less knowledge about their medications compared to those with regular prescriptions [37,38]. Many of our informants also described limited knowledge about their own medications, and more worrying some reported that the MDD service made it harder to track medications and reduced their sense of control. This is worrying, as initiation of the MDD service for patients who do not have problems with this part of medication management may lead to patients becoming more passive and less involved in their treatment, ultimately reducing their ability to self-manage medications [28,35,37,38].

However, not all users felt that MDD reduced their knowledge. One informant actually felt that their knowledge improved because the names of the medications were written on the MDD bags. This user was primarily interested in the names, and the MDD service made this information more accessible, empowering them to evaluate it. Our findings suggest that the lack of medication knowledge found in previous studies may be due to a decline in medication management capacity prior to MDD initiation. For patients with low interest or ability to evaluate information, MDD can provide much-needed support and relief, allowing them to feel more confident about correct dosing [28]. This can have a positive effect on autonomy and involvement. Therefore, a user’s initial interest and capacity to evaluate medication information are crucial factors in determining whether the MDD service empowers or enables self-management.

### Impact on the MDD Service and Policy

When MDD has been implemented in home care, it has been mostly seen as an aid to relieve the burden of dispensing and reduce medication errors [12]; however, this study shows that the service can affect all steps in medication management. It is thus important to consider the user’s individual preferences and routines to ensure safe medication use:

- There should be a standardized assessment of the patient’s medication management capabilities and motivation before starting MDD to help decide if MDD is the best solution for the patient or whether there are other forms of assistance that would be more beneficial. This assessment should identify which steps of medication management the patients have challenges with as well as to what degree they want to engage in the various steps.
- If MDD is deemed appropriate for the patient, the daily schedule of the patient should be noted so that the MDD bags can be adjusted accordingly (eg, making sure the time printed on the bags corresponds with the time the user gets up in the morning and have meals). This can help users making routines to remember to take medications.
- To ensure that patients have essential information about how to take their medications, the MDD service should make simplified medication information leaflets available to the patients. Patients should also be instructed to report to health care personnel if they experience side effects or other problems with their medication. In addition, home care workers should also have sufficient training to be able to educate and assist patients in understanding their medications better.
- For patients who have a low capacity to self-manage but still want to take an active part, the patients should be encouraged to make routines that provide a sense of control and flexibility (eg, routines for checking the contents of the MDD bags and allow for alterations of the bags under supervision).

### Strength and Limitations

This study is one of the few to examine how patients use adherence aids at home [12,17-19]. We included users from diverse socioeconomic backgrounds and with varying levels of home care assistance. However, it is important to note that the participants were all from one municipality, so the findings may not be easily applicable to other municipalities with different organization of home care services. It is also important to consider that patients who were very unhappy with the MDD service may have stopped using it, and their perspectives are not included in this study. Additionally, the voluntary nature of participation and recruitment by home care nurses may introduce a bias toward more empowered patients. Although the researchers have different educational background and age, both are female and Norwegian, and we discussed our sociocultural positions and value systems during the research process to try to limit the effect of preunderstanding bias.

### Conclusions

This study gives valuable insight into how the MDD service is used by home care users to support medication self-management. This study indicates that MDD can support users in their medication self-management and increase patient’s autonomy. However, the service does not support all phases of the medication process or support self-management for all patients. The degree to which MDD users manage their medications is not necessarily related to their actual capabilities for medication self-management. The patients’ own motivation for participating seems to be the most important factor affecting participation.

## Supplementary material

10.2196/57651 Multimedia Appendix 1 Interview guide.

## Abbreviations

COREQ: Consolidated Criteria for Reporting Qualitative Research
GP: general practitioner
MDD: multidose drug dispensing

## References

[1] Devik SA, Olsen RM, Fiskvik IL (2018). Variations in drug-related problems detected by multidisciplinary teams in Norwegian nursing homes and home nursing care. Scand J Prim Health Care.

[2] Sorensen L, Stokes JA, Purdie DM, Woodward M, Roberts MS (2006). Medication management at home: medication risk factor prevalence and inter-relationships. J Clin Pharm Ther.

[3] Kwint HF, Faber A, Gussekloo J, Bouvy ML (2012). The contribution of patient interviews to the identification of drug-related problems in home medication review. J Clin Pharm Ther.

[4] Lenander C, Elfsson B, Danielsson B, Midlöv P, Hasselström J (2014). Effects of a pharmacist-led structured medication review in primary care on drug-related problems and hospital admission rates: a randomized controlled trial. Scand J Prim Health Care.

[5] Brown MT, Bussell JK (2011). Medication adherence: WHO cares?. Mayo Clin Proc.

[6] Bailey SC, Oramasionwu CU, Wolf MS (2013). Rethinking adherence: a health literacy-informed model of medication self-management. J Health Commun.

[7] Marek KD, Antle L, Hughes RG (2008). Patient Safety and Quality: An Evidence-Based Handbook for Nurses.

[8] Sorensen L, Stokes JA, Purdie DM, Woodward M, Roberts MS (2005). Medication management at home: medication-related risk factors associated with poor health outcomes. Age Ageing.

[9] Holm SG, Mathisen TA, Sæterstrand TM, Brinchmann BS (2017). Allocation of home care services by municipalities in Norway: a document analysis. BMC Health Serv Res.

[10] (2019). Health at a glance 2019: OECD indicators. OECD Publishing.

[11] (2022). Pharmacy statistics (in Norwegian). Norwegian Pharmacy Association.

[12] Jøsendal AV, Bergmo TS, Granås AG, Olsen RM, Sletvold H (2022). Medication Safety in Municipal Health and Care Services.

[13] Rechel B (2018). Hub-and-spoke dispensing models for community pharmacies in Europe. Eurohealth (Lond).

[14] Sæther EM, Aandstad M, Hesthamar B (2007). Multidose drug dispensing: an economic assessment of measures.

[15] Sinnemäki J, Sihvo S, Isojärvi J, Blom M, Airaksinen M, Mäntylä A (2013). Automated dose dispensing service for primary healthcare patients: a systematic review. Syst Rev.

[16] Halvorsen KH, Granas AG (2012). Multi-dose dispensed drugs in Scandinavia—a systematic review of possibilities and limitations [in Norwegian]. Nor Farm Tidsskr.

[17] Boeni F, Spinatsch E, Suter K, Hersberger KE, Arnet I (2014). Effect of drug reminder packaging on medication adherence: a systematic review revealing research gaps. Syst Rev.

[18] Mahtani KR, Heneghan CJ, Glasziou PP, Perera R (2011). Reminder packaging for improving adherence to self-administered long-term medications. Cochrane Database Syst Rev.

[19] Mertz L, Tornbjerg K, Nøhr C (2021). User perception of automated dose dispensed medicine in home care: a scoping review. Healthcare (Basel).

[20] Schneider A, Körner T, Mehring M, Wensing M, Elwyn G, Szecsenyi J (2006). Impact of age, health locus of control and psychological co-morbidity on patients’ preferences for shared decision making in general practice. Patient Educ Couns.

[21] Náfrádi L, Nakamoto K, Schulz PJ (2017). Is patient empowerment the key to promote adherence? A systematic review of the relationship between self-efficacy, health locus of control and medication adherence. PLoS One.

[22] Osborne RH, Batterham RW, Elsworth GR, Hawkins M, Buchbinder R (2013). The grounded psychometric development and initial validation of the Health Literacy Questionnaire (HLQ). BMC Public Health.

[23] Holbø K, Das A, Bøthun S, Formanek MN, Halvorsen T (2019). Multidose service for home dwellers—the users’ experiences and a need for new solutions [in Norwegian]. Nord Welfare Res.

[24] Larsen AB, Haugbølle LS (2007). The impact of an automated dose-dispensing scheme on user compliance, medication understanding, and medication stockpiles. Res Social Adm Pharm.

[25] Robson C (2002). Real World Research.

[26] Braun V, Clarke V (2006). Using thematic analysis in psychology. Qual Res Psychol.

[27] Dowell J, Hudson H (1997). A qualitative study of medication-taking behaviour in primary care. Fam Pract.

[28] Nunney J, Raynor DK, Knapp P, Closs SJ (2011). How do the attitudes and beliefs of older people and healthcare professionals impact on the use of multi-compartment compliance aids?. Drugs Aging.

[29] Bell HT, Steinsbekk A, Granas AG (2017). Elderly users of fall-risk-increasing drug perceptions of fall risk and the relation to their drug use—a qualitative study. Scand J Prim Health Care.

[30] Ekdahl AW, Andersson L, Friedrichsen M (2010). “They do what they think is the best for me.” Frail elderly patients’ preferences for participation in their care during hospitalization. Patient Educ Couns.

[31] Levinson W, Kao A, Kuby A, Thisted RA (2005). Not all patients want to participate in decision making. J Gen Intern Med.

[32] Miller TA (2016). Health literacy and adherence to medical treatment in chronic and acute illness: a meta-analysis. Patient Educ Couns.

[33] Schulz PJ, Nakamoto K (2013). Health literacy and patient empowerment in health communication: the importance of separating conjoined twins. Patient Educ Couns.

[34] Hayes TL, Larimer N, Adami A, Kaye JA (2009). Medication adherence in healthy elders: small cognitive changes make a big difference. J Aging Health.

[35] Elliott RA (2014). Appropriate use of dose administration aids. Aust Prescr.

[36] Mertens BJ, Kwint HF, van Marum RJ, Bouvy ML (2018). Are multidose drug dispensing systems initiated for the appropriate patients?. Eur J Clin Pharmacol.

[37] Kwint HF, Stolk G, Faber A, Gussekloo J, Bouvy ML (2013). Medication adherence and knowledge of older patients with and without multidose drug dispensing. Age Ageing.

[38] Modig S, Kristensson J, Ekwall AK, Hallberg IR, Midlöv P (2009). Frail elderly patients in primary care—their medication knowledge and beliefs about prescribed medicines. Eur J Clin Pharmacol.

