# Supplementary material for: Medication Self-Management for Home Care Users Receiving Multidose Drug Dispensing: Qualitative Interview Study
Source: JMIR Aging. 2024 Oct 4;7:e57651. doi: 10.2196/57651 (PMC11468972; doi:10.2196/57651)
Supplement: Multimedia Appendix 1 [file aging-v7-e57651-s001.pdf]

Interview guide

Introduction

- Repetition of aim of the study, voluntary participation and the opportunity to withdraw consent. Signing of informed consent form.
- Estimated time of interviews
- Audiotape
- Display the multidose bags and other medications

Medication use and management

- How do you feel about taking medicines?
- Do you manage the medications yourself or receive help. If so, by who and with what? (also include renewing prescriptions and collecting medications)
- How long have you used multidose?
- How did you manage your medication before you got MDD?
- How do you feel about the multidose system (opening the bags, remembering to take them, keeping track of medications)
- What challenges do you experience in your medication management, if any?
- Do you ever forget to take your medications or decide not to take certain medications? If so, how often or in what situations?
- Do you have any thoughts on how your medication management can be done in a safer or easier way?

Medicines information

- How do you keep track of the medicines you are using?
- Do you know why you are taking these medications?
- What information did you get when starting medications, and by whom? (written or oral information)
    - Have you read the information you've received? Why/why not?
- Where do you generally get information about your medications from (both those in MDD and those in their original packaging)

    - Do you use computers or the internet to look up medicines information?
- If you experience any problems or have questions about your medications, what do you do?
- How were you involved when multidose was initiated?
    - To what extent did you find the information sufficient?
- Do you feel that the multidose system has changed your way of keeping track of medicines. If so in what way?

End of interview:

- Sum up
- Something more to add?
- Thank you for your time

**Notes**	
Age and gender	
Use of medications, MDD, dietary supplements, doesette boxes<br>Storing of medications	
Other	
